# Optimizing huddle engagement through leadership and problem-solving within primary care: A study protocol for a cluster randomized trial

**DOI:** 10.1186/s13063-018-2847-5

**Published:** 2018-10-04

**Authors:** Megan E. Branda, Aravind Chandrasekaran, Marc D. Tumerman, Nilay D. Shah, Peter Ward, Bradley R. Staats, Theresa M. Lewis, Diane K. Olson, Rachel Giblon, Michelle A. Lampman, David R. Rushlow

**Affiliations:** 1 Division of Health Care Policy and Research, Department of Health Sciences Research, Mayo Clinic, 200 First Street SW, Rochester, MN 55905 USA; 2 Robert D. and Patricia E. Kern Center for the Science of Health Care Delivery, Mayo Clinic, Rochester, MN USA; 3 Knowledge and Evaluation Research Unit, Divisions of Endocrinology and Diabetes, Mayo Clinic, Rochester, MN USA; 4 Center for Operational Excellence, Fisher College of Business, The Ohio State University, Columbus, OH USA; 5 Department of Family Medicine, Mayo Clinic, Sparta, WI USA; 6 Kenan-Flagler Business School, University of North Carolina, Chapel Hill, NC USA; 7 Public Affairs, Mayo Clinic Health System, Rochester, MN USA; 8 Mayo Clinic Health System Franciscan Healthcare, La Crosse, WI USA

**Keywords:** Team-based care, Huddle, Training, Primary care

## Abstract

**Background:**

Team-based care has been identified as a key component in transforming primary care. An important factor in implementing team-based care is the requirement for teams to have daily huddles. During huddles, the care team, comprising physicians, nurses, and administrative staff, come together to discuss their daily schedules, track problems, and develop countermeasures to fix these problems. However, the impact of these huddles on staff burnout over time and patient outcomes are not clear. Further, there are challenges to implementing huddles in fast-paced primary care clinics. We will test whether the impact of a behavioral intervention of leadership training and problem-solving during the daily huddling process will result in higher consistent huddling in the intervention arm and result in higher team morale, reduced burnout, and improved patient outcomes.

**Methods/design:**

We will conduct a care-team-level cluster randomized trial within primary care practices in two Midwestern states. The intervention will comprise a 1-day training retreat for leaders of primary care teams, biweekly sessions between huddle optimization coaches and members of the primary care teams, as well as coaching site visits at 30 and 100 days post intervention. This behavioral intervention will be compared to standard care, in which care teams have huddles without any support or training. Surveys of primary care team members will be administered at baseline (prior to training), 100 days (for the intervention arm only), and 180 days to assess team dynamics. The primary outcome of this trial will be team morale. Secondary outcomes will assess the impact of this intervention on clinician burnout, patient satisfaction, and quality of care.

**Discussion:**

This trial will provide evidence on the impact of a behavioral intervention to implement huddles as a key component of team-based care models. Knowledge gained from this trial will be critical to broader deployment and successful implementation of team-based care models.

**Trial registration:**

Clinicaltrials.gov, NCT03062670. Registered on 23 February 2017.

**Electronic supplementary material:**

The online version of this article (10.1186/s13063-018-2847-5) contains supplementary material, which is available to authorized users.

## Background

The Centers for Medicare and Medicaid Services (CMS) has made population health management a key priority as part of its efforts to transform primary care delivery [1]. In 2016, CMS announced $157 million in funding to reform the delivery of primary care to improve both quality and affordability. Research shows that the current approach to delivering primary care using a provider-centric approach is ineffective and may not allow health systems to pursue simultaneously the triple goals of better population health, better patient experiences, and lower per capita costs [2]. There is a need to innovate the primary care delivery model and transition it to a team-based model from a provider-centered care delivery approach. Team-based care has been identified as a key element in transforming primary care [3]. Although theoretical arguments exist on the importance of team-based care in transforming primary care, there is lack of empirical evidence on how team-based care models affect primary care outcomes [4]. With this lack of evidence, the adoption of this model into routine primary care practices has been slow [3]. Making the shift from provider-centric to team-based care has produced high-performing teams when training is one of the components of the implementation [5]. Team training has been found to be useful for improving teamwork, performance, and cognitive and affective outcomes [6]. Team-based care has been endorsed by the Agency for Healthcare Research and Quality (AHRQ) and it now provides online training for implementing team-based care within health-care settings [7]. Within primary care settings, adopting a team-based approach can enhance communication and improve efficiency, particularly with the use of daily huddles.

AHRQ recommends that teams should huddle every morning for at least 10 min [7]. During huddles, the care team, comprising physicians, nurses, and administrative staff, come together to discuss their daily schedules, track problems, and develop countermeasures to fix these problems [8]. Bringing everyone together more frequently has the potential to minimize hierarchical barriers during the delivery of care [9]. Huddles have also been shown to be associated with higher satisfaction of front-line staff and improved communication among the care team [10].

However, many challenges to implementing huddles are also noted in the literature. In a study of six Veterans Health Administration primary care practices that involved over 400 care team members, Rodriguez et al. found that facilitating and encouraging leadership are some of the critical facilitators to daily huddling [11]. Training the physicians to be effective leaders during a huddle can help sustain the practice of huddles [12].

There is very limited evidence on the effectiveness of huddles in enhancing physician engagement in team-based care and problem-solving in outpatient care models. While a few studies argue for the importance of daily problem-solving, these studies are conceptual or based on limited empirical evidence. The gap between the conceptual evidence and successful real-world implementation is limiting our understanding of the impact and the adoption into practice [6, 13].

### Objectives

Through this research we intend to evaluate whether a behavioral intervention of leadership training and problem-solving during the daily huddle will result in better team morale, reduced burnout, and improved patient satisfaction and outcomes over the standard implementation of huddles. Our hypothesis is that the proposed intervention will lead to improved outcomes in a primary care setting.

## Methods/design

This is a multicenter parallel cluster randomized trial in which we test the superiority of huddle optimization training versus standard practice in huddles. Assignment to arms was in a 1–1 ratio. Institutional Review Board (IRB) approval has been obtained from the coordinating center, the Mayo Clinic (approval 16–010146). The trial is registered at clinicaltrials.gov (registration number NCT03062670, registration date 23 February 2017). Our protocol adheres to the SPIRIT recommendations (see Additional file 1) [14].

Primary care clinics in the Midwest United States that are within the Mayo Clinic Health System were eligible for participation if they were certified by the health system as providing team-based care and implementing daily huddles. Care teams in primary care internal medicine, family medicine, or pediatrics were eligible. Care teams seeking enrollment were required to commit to team-based care and implement daily huddles. Within each practice site, care teams were asked to appoint an administrator, a lead physician, and a lead nurse who would attend the huddle optimization training in the event their team was randomized to the intervention arm. All members of the care team who attend huddles were eligible for inclusion and were asked to complete the surveys. Each member has the option to opt out of the surveys.

### Intervention

The intervention comprised a full-day retreat on leadership development, team-based care development, and problem-solving with a focus on huddles as the key strategy. The training at the retreat was provided by experts from the Leadership and Organizational Development Program at Mayo Clinic. Each care team was represented by a physician, a nurse and a clinic administrator.

The retreat consisted of the following learning objectives:

- Leadership development focusing on physician engagement and teamwork
- Effective huddling as a leadership practice
- Problem-solving focusing on huddles and visual management

Upon completion of the retreat, the research team supported the initiation of daily huddles in the teams randomized to the intervention arm. Other care providers, including pharmacists, receptionists, social workers, and behavioral interventionists, were invited to join the huddles. Each of the intervention teams was assigned to one of the 6 coaches, who provided help and resources during the study period. Biweekly calls, when possible, were established between the coaches and a lead within each care team. There were also coaching visits (at 30 and 100 days) to the individual research sites upon the implementation of these huddles. The content of the coaching was standardized across the treatment sites and primarily involved the lessons learned during the retreat. Specifically, the coaching visits had the following objectives:

- 30-day visit (first interaction): Sustaining the huddling experience
- 100-day visit (second interaction): Effectiveness of problem-solving in huddles and innovation in team-based care

During these visits, there was time for additional interactions with the huddling members and to assess the huddles.

### Standard care

Current standard care varied among the teams. While the huddles were to happen daily, the actual implementation depended on the team. Teams not randomized to the intervention were provided with self-directed training materials related to implementing team-based care, which included an article on the use of huddles developed by Rodriquez et al. [11]. Each team was expected to use these materials to form and conduct huddles per their understanding and their unique needs.

### Outcomes

The primary outcome for the trial is a previously tested and validated team measurement score that consists of 31 questions, asking the respondent to answer “agree” or “disagree” on a four point scale that is converted to a 100 point score per methods referenced in Stock et al. [15]. The team measurement score focuses on four elements of team behavior: communication, roles and goals, cohesion, and team primacy. Responses from each team member will be used to generate an overall score for the team.

Secondary aims include patient satisfaction assessed through Press Ganey ® Medical Practice surveys. These surveys are implemented routinely within the health-care system. After a patient has had an appointment, if they are randomly selected, the survey is sent to them within 30 days for completion. A random sample of each team’s assigned patients that had a recent appointment were surveyed by Press Ganey® every quarter and reported at the site, care team, and provider levels. Patients’ responses are de-identified so that we know who provided the care but not to whom. We receive the percentage of patients who rated their provider with a top score for a given quarter. We monitor the quality outcomes in chronic disease management and prevention to ensure safety and an adequate quality of care are provided in both the control and intervention arms. Quality metrics at the care team level are assessed monthly for diabetes, depression, asthma, hypertension, and vascular issues. The metrics are calculated as the number of patients who met the quality criteria divided by the number of patients who are assigned to the care team who qualify for the assessment. The criteria for each metric are set by Minnesota Community Measures [16].

### Participant timeline

**Fig. 1:**
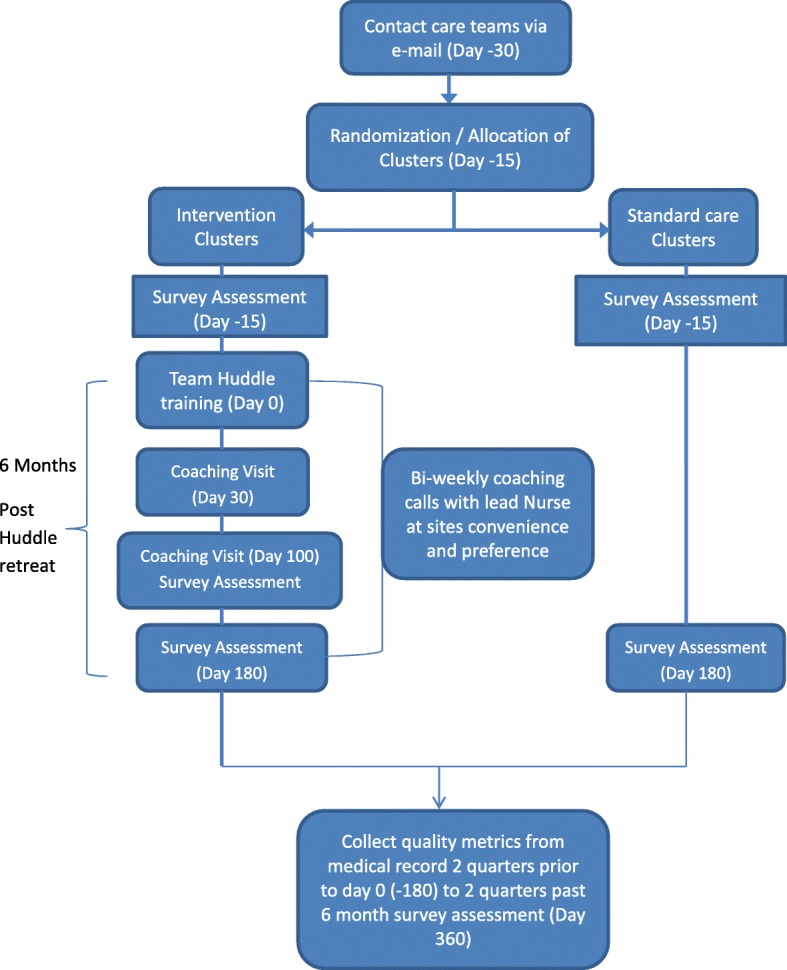
Study flow diagram

Participation in the study for the care teams ended when the last survey was administered at 6 months post the start of the intervention (Fig. 1). Further data will be collected from medical records at the care team level up to 12 months after the end of the intervention, but no contact with the participants will be needed.

### Sample size

Assuming 10 clusters per arm with an intra-cluster correlation of 0.05, with a minimum of three participants per cluster, we would be powered at 90% to detect a 1 standard deviation difference between continuous outcomes. If the cluster size is 10 individuals, then we would be powered at 80% to detect a half standard deviation difference between two continuous outcomes. All calculations are based on a two-sided test with an alpha of 0.05. To ensure there was adequate support for the retreat and that the coaches can support the teams, a maximum of 15 care teams can be randomized into the intervention arm.

### Recruitment

All sites that meet the eligibility criteria were contacted via email for participation in the trial. All administrators, physicians, and nurses were sent the email asking for a representative of the care team to contact the study team with consent to participate. The email, with text approved by the IRB, was used as the invitation for participation and consent to participate (Additional file 2).

### Allocation

Groups of teams or a team were randomized in a 1–1 allocation between the intervention and control groups. The groups were balanced across type (primary care internal medicine, family medicine, and pediatrics) and panel size (large vs. other). A computer algorithm accessed by the study statistician randomized the groups using dynamic allocation [17]. Blinding did not occur.

Concerns about contamination did not allow for randomization at the care team level as staff within a care team could overlap with other sites and other care teams within a site. After recruitment had completed, the study team statistician assessed the staff overlap and grouped the teams based on this to ensure that each unit of randomization was unique.

### Methods for data collection

Self-reported responses from staff within the enrolled care teams attending the huddles were collected at baseline and 6 months post enrollment. The intervention group completed the survey at 100 days as well. The survey is the same for each time point with the exception of the 6-month survey, which also had questions to assess burnout [18]. The surveys were sent via email, with up to three reminders for non-responders.

For the quality metrics, the data were from aggregated medical records for all patients assigned to the care team. The time frame for collection was from 6 months prior to the start of the study to 12 months post (6 months after the end of the intervention period). The metrics are described in Section “Outcomes”.

The following measures were collected pre-intervention, after 100 days (intervention only), and after 180 days (Additional file 3). The scales collected include team cohesion, psychological safety, and team autonomy. Team cohesion captures the extent to which members of the care team stick together in terms of helping each other and defending each other from criticism. It was measured using a four-item scale adapted from Lewis [19]. Psychological safety measures the extent to which care team members can freely express their opinions without negative repercussions. It is defined as “a shared belief that the team is safe for interpersonal risk taking.” Psychological safety is measured using a six-item scale developed by Edmondson [20]. Team autonomy measures the degree of involvement of the care team members in determining huddle goals, performance evaluation, and task assignment. A five-item scale adapted from Thompson was used to measure autonomy [21]. For staff demographics, we collected gender and provider role.

### Data management

Survey data were collected by the Survey Research Center, which is an institutional resource for conducting mail, web, and telephone surveys. The Survey Research Center works closely with Mayo Clinic’s IRB to ensure compliance with legal requirements and Mayo Clinic’s policies for surveys. All Survey Research Center staff receive IRB and HIPAA training, testing, and supervision to ensure the information dealt with is treated confidentially and consistent with Mayo policy. Data captured from the assigned patients at the care team level will be stored within the Mayo Clinic system, which is password protected and backed up every night. Press Ganey ® patient satisfaction surveys are administered via mail by Mayo Clinic. Mayo Clinic statistical study team members have access to study data.

### Statistical methods

All teams will be analyzed in the arm to which they were assigned using techniques appropriate for pragmatic cluster randomized trials. Baseline characteristics will be summarized at the site and care team levels within each trial arm, to provide counts and frequencies for categorical variables and means with standard deviations and ranges for continuous variables. We will test the null hypothesis of no difference between arms in baseline characteristics using *t*-tests and chi-square tests adjusted for clustering by care team.

To account for the effects of care team clustering, we will use hierarchical generalized linear models with random main effects specified at the care team level. Such models are appropriate for analyzing cluster randomized trials and will allow us to account for any imbalances in care team characteristics across study arms. For measures collected from the team members, the outcome of interest will be the 6-month post-baseline responses, adjusting for baseline responses. If there are differences in baseline characteristics between the two study groups, these will be accounted for using hierarchical generalized logistic or linear regression models that include an indicator for study arm.

For outcomes collected at the care team level (patient satisfaction and quality measurements), the percentage of patients who gave the top score for satisfaction and the percentage of patients who met the quality criteria will be compared between arms without accounting for clustering effects, as the data are not available at the individual level.

### Missing data

We will make every effort to minimize the amount of missing data. Trial enrollment and the fidelity of follow-up procedures will be reviewed during biweekly conference calls. A study biostatistician will produce frequency reports to assess for missing data, and the study team will troubleshoot any problems encountered. We will report the rates of missing data for each outcome by study arm.

### Safety and monitoring

This study does not pose any potential harm to care team staff or their patients and all current procedures at the sites for the care teams will continue to be used. All participating staff have given consent. They may withdraw at any time with no impact on their position in the health system.

## Discussion

Transforming primary care delivery is a key priority in the United States for addressing population health management. Providing care with a team-based approach has been proposed and evaluated as a solution for primary care. To the best of our knowledge, there is no empirical evidence on the role of team-based care in improving patient safety, patient centeredness, and health outcomes. The formation of a team alone cannot guarantee success. There are many methodological proposals on proper team implementation and some include training for the team members. We propose that giving the leaders (physicians, nurses, and administrators) skills they can implement within the daily huddles of their care teams, concentrating specifically on communication and problem-solving skills, will directly impact and improve the quality of teamwork.

Our aim is to evaluate the effectiveness of our intervention on team morale, clinician burnout, patient satisfaction, and quality of care. Our cluster randomized trial has been designed to test the impact of the training on leadership and problem-solving skills within daily huddles in the context of primary care. Our inclusion criteria cover many sites meeting the needs of a broad range of populations.

This trial is being implemented within routine care. The demands of busy practices may cause logistical problems. The ability of each care team to continue to huddle every day and the participation of the intervention arm in the training and the continued activities of the intervention (biweekly phone calls with a trainer and the visit after 100 days by a trainer) may differ by care team. Staff turnover within primary care causes additional challenges, in that the team dynamic is affected along with loss of information and time that goes into training new personnel.

The evaluation of the outcomes of the trial will allow us to explore the impact of the training and the ability of the care teams to implement huddles within primary care. If the intervention is proven to be successful, it will be implemented in standard care sites on their request. The implementation strategy for the intervention will be assessed in this setting, as we will need to assess the impact on the care teams. All study results will be presented to the Mayo Clinic Health System on completion and the results will be published.

### Trial status

In total, 30 care teams responded to the email invitation to participate. The 30 care teams consisted of 23 unique randomizing units due to staff overlap. Post-randomization prior to the baseline surveys being sent out, one unit that had one care team and another two care teams withdrew from participation. Excluding the care team that withdrew, 11 units were randomized to the intervention arm (13 care teams) and 11 to usual care (16 care teams). The retreat took place on 13 April 2017. Data collection is still ongoing.

## Additional files

Additional file 1: SPIRIT checklist. (DOC 135 kb)

Additional file 2: Clinician contact email for participation and consent to participate. (DOCX 25 kb)

Additional file 3: Clinician survey. (DOCX 33 kb)

## Abbreviations

AHRQ: US Agency for Healthcare Research and Quality
CMS: Centers for Medicare and Medicaid Services
IRB: Institutional Review Board
