# Supplementary material for: Optimizing huddle engagement through leadership and problem-solving within primary care: A study protocol for a cluster randomized trial
Source: Trials. 2018 Oct 4;19:536. doi: 10.1186/s13063-018-2847-5 (PMC6172734; doi:10.1186/s13063-018-2847-5)
Supplement: Supplementary file 2 — Clinician contact email for participation and consent to participate. (DOCX 25 kb) [file 13063_2018_2847_MOESM2_ESM.docx]

## Message to Care Teams- Huddle Research Project

**From:** Dr. Dave Rushlow

**To:** Individual primary care physicians, nurse leaders, and CAOs **Cc:** Huddle Project Research team (use list from Aravind), plus Dr. Kari Bunkers, Dr. Rob Stroebel, Kris Johnson, Lesa Anderson, Nilay Shah, Theresa Lewis

**Email Subject Line:** Practice-based research opportunity for MCHS primary care teams

**Text for email message:**

We are pleased to offer Mayo Clinic Health System (MCHS) primary care teams a unique opportunity to participate in a clinical research project. The Huddle Research Project will seek to demonstrate the effectiveness of team-based care by utilizing daily huddles. Please understand your participation is voluntary and you have the right to withdraw your consent or discontinue participation at any time without penalty. Specifically, your current or future medical care and employment at Mayo Clinic will not be jeopardized if you choose not to participate.

**A high level overview of the research opportunity:**

**Who:** Primary Care Teams in MCHS

**What:** Opportunity to volunteer to participate as a care team in the Huddle Research Project

**Why:**

- Enhance care team development and efficiency
- Increase provider and staff satisfaction
- Improve patient/quality outcomes

**When:**

- Study participant primary care team requests to participate will be accepted through <insert date>
- The study will take place from April to September 2017

**How:**

1. As a primary care team, make a decision to volunteer for the study.
2. Have one care team leader respond to this email with:
   1. “Yes” vote for your team (using the Outlook voting buttons)
   2. A list the names of all members on your primary care team
3. Be committed to training and coaching as required by the study for intervention groups
   (Note: each care team chosen for the intervention will be required to have representation at a half-day retreat from the roles of a physician and nurse from the same team, and a clinic administrator/site leader.)
4. As a primary care team, participate in the intervention group or control group, as randomly assigned.

**Details about the Huddle Research Project**

On December 21, Midwest Community Care Clinical Practice Committee (MWC CPC) approved the Huddle Research Project for Mayo Clinic Health System (MCHS). The study will be primary care-focused and coincide with the Mayo Model of Community Care (MMoCC) team-based care principles. Dave Rushlow, M.D., CMO SWWI Region and principle investigator for the study, proposed the MCHS practice-based research project to enhance team-based care by utilizing structured huddles.

Before proposing the study, Dr. Rushlow and the research team performed site visits, conducted interviews with primary care providers, and observed care team huddles.

The preliminary research revealed variations in:

- Approaches to delivering team-based care
- Care team participants involved in huddles
- Focus of huddle discussions
- Buy-in and engagement of care team members
- Quality and patient experience measures

The findings lead the research team to recommend an investigation of the factors that may enhance team-based care, as well as, improve provider/staff satisfaction, and patient outcomes. The Huddle Research Study will experiment with a subset of primary care teams to demonstrate the effectiveness of team-based care through daily huddles. The study will involve randomly assigning intervention and control groups from a pool of care teams who volunteer for the study. The intervention will comprise of a full-day retreat in mid-March. The retreat will focus on huddles as the key strategy and incorporating training for leadership development, team-based care development, and problem solving. Each primary care team at the retreat will be represented by a physician and nurse (from the same team), plus a clinic administrator/site leader. In addition to the retreat, coaching sessions will occur with each care team at 30 and 120 days. The intervention period will last 6 months.

The retreat’s learning objectives:

- Leadership development focusing on teamwork and engagement
- Effective huddling as a leadership practice
- Problem solving focusing on huddles and visual management

Upon the completion of the retreat, the treatment group will initiate the daily huddles in the respective treatment sites that will involve primary care providers and allied health staff, and, if applicable, pharmacists, receptionists, social workers and behavioral interventionists. The treatment group will also be provided with an opportunity to avail any help and resources from the researchers at Mayo during the study period.

To understand how the team-based measures change over time, the research team will conduct pre-assessments and periodic assessments of outcomes. The hypothesis is that the treatment group will perform better on the outcomes when compared to the control group. The research team will evaluate whether a team-based care delivery approach using daily huddles is effective in improving provider/staff satisfaction, and patient outcomes in the primary care setting. This approach is expected to not only lead to effective primary care delivery outcomes, but also result in greater team morale, reduced burnout, and a higher rate of innovation.

Volunteer care teams are now being sought to participate in the Huddle Research Project. After the enrollment process, the volunteer care teams will be randomly assigned to treatment and control groups with a maximum of 15 teams for the treatment group. Upon completion of the study in 6 months, the control group will be offered the intervention, with iterative improvements based on the findings.

If your care team makes the decision to enroll in the Huddle Research Project, please have one care team leaders respond to this email with a “Yes” vote (via the Outlook voting button) and list the names of all primary care team members.

Please let me know if you have any questions.

Thank you.

Dave Rushlow, M.D., Huddle Research Project Principle Investigator
