# Supplementary material for: Optimizing huddle engagement through leadership and problem-solving within primary care: A study protocol for a cluster randomized trial
Source: Trials. 2018 Oct 4;19:536. doi: 10.1186/s13063-018-2847-5 (PMC6172734; doi:10.1186/s13063-018-2847-5)
Supplement: Supplementary file 3 — Clinician survey. (DOCX 33 kb) [file 13063_2018_2847_MOESM3_ESM.docx]

SUBJECT LINE: Huddle Research Project

Dear <lastname>,

You have recently agreed to participate in the Huddle Research Project (IRB Approval # 16-010146). This research project is seeking to demonstrate the effectiveness of team-based care by using enhanced daily huddles through enhancing team cohesion, developing leadership skills and collaboration.

As a part of the study, please take a few minutes to complete the survey. Your responses are confidential and will not be shared with your team or clinic. If you have any questions or comments about this survey or study, please call: 1-507-xxx-xxxx. If you should encounter technical problems in filling out the survey, please contact the Survey Research Center ([surveyresearchcenter@mayo.edu](mailto:surveyresearchcenter@mayo.edu)).

To complete the survey, click on “Take Survey” below:

<TAKE SURVEY LINK>

Sincerely,

David Rushlow, MD

Huddle Research Project Principal Investigator

Q1 **HUDDLE OPTIMIZATION STUDY**
**(aka HUDDLE RESEARCH PROJECT)**

Q2
**Please rate the following statements based on the interactions your team had during your most recent huddling activity.**

|  | Strongly disagree (1) | Somewhat disagree (2) | Neither agree nor disagree (3) | Somewhat agree (4) | Strongly agree (5) |
| --- | --- | --- | --- | --- | --- |
| **Our team worked together in a well-coordinated fashion.** (1) |  |  |  |  |  |
| **Our team had very few misunderstandings about what to do.** (2) |  |  |  |  |  |
| **Our team needed to backtrack and start over a lot.** (3) |  |  |  |  |  |
| **We accomplished the task smoothly and efficiently.** (4) |  |  |  |  |  |
| **There was much confusion about how we would accomplish the task.** (5) |  |  |  |  |  |
| **I was comfortable accepting procedural suggestions from other team members.** (6) |  |  |  |  |  |
| **I trusted that other members' knowledge about the activity was credible.** (7) |  |  |  |  |  |
| **I was confident relying on the information that other team members brought to the discussion.** (8) |  |  |  |  |  |
| **When other members gave information, I wanted to double-check it for myself.** (9) |  |  |  |  |  |
| **I did not have much faith in other members' "expertise."** (10) |  |  |  |  |  |

Q3
**Please rate the following statements regarding the team climate during your most recent huddling activity.**

|  | Strongly disagree (1) | Somewhat disagree (2) | Neither agree nor disagree (3) | Somewhat agree (4) | Strongly agree (5) |
| --- | --- | --- | --- | --- | --- |
| **If you make a mistake on this team, it is often held against you.** (1) |  |  |  |  |  |
| **Members of this team are able to bring up problems and tough issues.** (2) |  |  |  |  |  |
| **People on this team sometimes reject others for being different.** (3) |  |  |  |  |  |
| **It is safe to take a risk on this team.** (4) |  |  |  |  |  |
| **It is difficult to ask other members of this team for help.** (5) |  |  |  |  |  |
| **No one on this team would deliberately act in a way that undermines my efforts.** (6) |  |  |  |  |  |
| **Working with members of this team, my unique skills and talents are valued and utilized.** (7) |  |  |  |  |  |

Q4
**Please rate the following statements regarding the team decision-making and empowerment levels during your most recent huddling activity.**


**Our team had authority to accomplish the following tasks during our daily huddles:**

|  | Strongly disagree (1) | Somewhat disagree (2) | Neither agree nor disagree (3) | Somewhat agree (4) | Strongly agree (5) |
| --- | --- | --- | --- | --- | --- |
| **Planning and determining goals** (1) |  |  |  |  |  |
| **Decisions concerning leadership within the team** (2) |  |  |  |  |  |
| **Performance evaluation of the team** (3) |  |  |  |  |  |
| **Task assignments within the team** (4) |  |  |  |  |  |

Q5
**Please rate the following statements regarding the learning that occurred during your most recent huddling activity.**

|  | Strongly disagree (1) | Somewhat disagree (2) | Neither agree nor disagree (3) | Somewhat agree (4) | Strongly agree (5) |
| --- | --- | --- | --- | --- | --- |
| **Being a part of this team has been a great learning experience for the team members.** (1) |  |  |  |  |  |
| **Team members' experience with the work is likely to help them perform better on work in the future.** (2) |  |  |  |  |  |
| **Team members are likely to repeat the mistakes made here on other work.** (3) |  |  |  |  |  |
| **Members are likely to apply the lessons learned on this work to other areas in the organization.** (4) |  |  |  |  |  |

Q6
**Please rate the following statements regarding knowledge generated during your most recent huddling activity.**

|  | Strongly disagree (1) | Somewhat disagree (2) | Neither agree nor disagree (3) | Somewhat agree (4) | Strongly agree (5) |
| --- | --- | --- | --- | --- | --- |
| **Doing huddles enhanced the team's abilities and knowledge to perform future work.** (1) |  |  |  |  |  |
| **Solutions found during this huddling activity were clearly unique and innovative for our clinic.** (2) |  |  |  |  |  |
| **Our team generated several ideas during the huddling activity.** (3) |  |  |  |  |  |

Q7
**This questionnaire is a measure of team characteristics.  Please indicate how much you strongly disagree, disagree, agree, or strongly agree to each statement as it applies to your team at the present time.  There are no right or wrong answers, just your perceptions.**

|  | Strongly disagree (1) | Disagree (2) | Agree (3) | Strongly agree (4) |
| --- | --- | --- | --- | --- |
| **Team members say what they really mean.** (1) |  |  |  |  |
| **Team members say what they really think.** (2) |  |  |  |  |
| **Team members talk about other team members behind their back.** (3) |  |  |  |  |
| **All team members participate in making decisions about the work of the team.** (4) |  |  |  |  |
| **All team members feel free to share their ideas with the team.** (5) |  |  |  |  |
| **All team members feel free to express their feelings with the team.** (6) |  |  |  |  |
| **The team practices tolerance, flexibility, and appreciation of the unique differences between team members.** (7) |  |  |  |  |
| **The team handles conflicts in a calm, caring, and healing manner.** (8) |  |  |  |  |
| **Regardless of the topic, communication between the people on this team is direct, truthful, respectful, and positive.** (9) |  |  |  |  |
| **The team openly discusses decisions that affect the work of the team before they are made.** (10) |  |  |  |  |

Q8 **Continued next page...**

Q9
**Continued...**
 
**Please indicate how much you strongly disagree, disagree, agree, or strongly agree to each statement as it applies to your team at the present time.  There are no right or wrong answers, just your perceptions.**

|  | Strongly disagree (1) | Disagree (2) | Agree (3) | Strongly agree (4) |
| --- | --- | --- | --- | --- |
| **In this team, members support, nurture, and care for each other.** (1) |  |  |  |  |
| **The team has agreed upon clear criteria for evaluating the outcomes of the team's effort.** (2) |  |  |  |  |
| **As a team we come up with creative solutions to problems.** (3) |  |  |  |  |
| **In the team there is more of a WE feeling than ME feeling.** (4) |  |  |  |  |
| **There is confusion about what the work is that the team should be doing.** (5) |  |  |  |  |
| **There is confusion about how to accomplish the work of the team.** (6) |  |  |  |  |
| **Roles and responsibilities of individual team members are clearly understood by all members of the team.** (7) |  |  |  |  |
| **All team members place the accomplishments of the team ahead of their own individual accomplishments.** (8) |  |  |  |  |
| **The goals of the team are clearly understood by all team members.** (9) |  |  |  |  |
| **All team members define the goals of the team as more important than their own personal goals.** (10) |  |  |  |  |

Q10 **Continued next page...**

Q11
**Continued...**
 
**Please indicate how much you strongly disagree, disagree, agree, or strongly agree to each statement as it applies to your team at the present time.  There are no right or wrong answers, just your perceptions.**

|  | Strongly disagree (1) | Disagree (2) | Agree (3) | Strongly agree (4) |
| --- | --- | --- | --- | --- |
| **I am happy with the outcomes of the team's work so far.** (1) |  |  |  |  |
| **I enjoy being in the company of the other members of the team.** (2) |  |  |  |  |
| **Being on this team is a personally meaningful experience for me.** (3) |  |  |  |  |
| **I have a clear understanding of what other team members expect of me as a team member.** (4) |  |  |  |  |
| **The work I do on this team is valued by the other team members.** (5) |  |  |  |  |
| **I am allowed to use my unique personal skills and abilities for the benefit of the team.** (6) |  |  |  |  |
| **Some members of this team resist being led.** (7) |  |  |  |  |
| **Information that is important for the team to have is openly shared by and with all team members.** (8) |  |  |  |  |
| **All individuals on this team feel free to suggest ways to improve how the team functions.** (9) |  |  |  |  |
| **When team problems arise the team openly explores options to solve them.** (10) |  |  |  |  |
| **On this team, the person who takes the lead differs depending on who is best suited for the task.** (11) |  |  |  |  |

Q12 <180 day only>

**Following are 2 statements of job-related feelings.  Please read each statement carefully and decide if you ever feel this way *about your job*.  If you have never had this feeling, mark "Never."  If you have had this feeling, indicate *how often* you feel it by marking the response (0 to 6) that best describes how frequently you felt that way.**

|  | Never    0 (0) | A few times a year or less    1 (1) | Once a month or less    2 (2) | A few times a month    3 (3) | Once a week    4 (4) | A few times a week    5 (5 | Every day    6 (6) |
| --- | --- | --- | --- | --- | --- | --- | --- |
| **I feel burned out from work.** (1) |  |  |  |  |  |  |  |
| **I've become more callous toward people since I took this job.** (2) |  |  |  |  |  |  |  |

Q13 **Do you have any other comments and/or feedback regarding the huddling activity in your site?**

________________________________________________________________

________________________________________________________________

________________________________________________________________

________________________________________________________________

________________________________________________________________

Q14
**Thank you for your time!

 Please click SUBMIT to record your responses.**
